# Colonization and extinction dynamics among the plant species at tree bases in Paris (France)

**DOI:** 10.1002/ece3.4954

**Published:** 2019-07-18

**Authors:** Mona Omar, Laure Schneider‐Maunoury, Kévin Barré, Nazir Al Sayed, Jalal Halwani, Nathalie Machon

**Affiliations:** ^1^ Centre d'Ecologie et des Sciences de la Conservation (CESCO), Muséum National d'Histoire Naturelle, Centre National de la Recherche Scientifique Sorbonne Universités Paris France; ^2^ Water & Environment Science Laboratory, Faculty of Public Health Lebanese University Tripoli Lebanon; ^3^ Institut de Systématique, Évolution, Biodiversité (ISYEB), Muséum National d'Histoire Naturelle, Centre National de la Recherche Scientifique Sorbonne Universités Paris France; ^4^ Faculty of Engineering, Section I Lebanese University Tripoli Lebanon

**Keywords:** Levins’ model, metapopulation, propagule rain model, rescue effect, seed longevity, SPOMSIM, spontaneous flora, urban biodiversity

## Abstract

1. In cities, trees planted along streets could play an important ecological role for spontaneous plants growing at their bases. For example, these trees could represent corridors by potentially connecting large green spaces (e.g., parks, gardens), which allow species to move within the urban matrix. We considered sets of urban trees in 15 streets in Paris, France, as metapopulations for 15 plant species. Our objective was to determine the factors influencing the dynamics of colonization and extinction of populations based on the distance of the streets to green spaces and biological traits of each species.
2. Plant species in 1,324 tree bases of the Bercy District of Paris were surveyed annually from 2009 to 2015. For each species and each street, we used SPOMSIM software to identify the best‐fit metapopulation model between four models with different colonization and extinction functions: propagule rain model (PRM) and Levins’ model with or without rescue effect.
3. Results demonstrated that species more often conformed to the PRM in streets near green spaces, which suggested that green spaces could act as sources for the populations in those streets. Species with seeds with long‐term persistence more often conformed to the PRM, indicating that a soil seed bank helps species invade entire streets. Finally, a higher percentage of species with a short height conformed to models with a rescue effect, which indicated that those small species resisted the effects of weeding by the city technical services better than taller species.
4. Synthesis and applications. This study showed how biological traits of species and geography of the district determine the dynamics of plants in the streets, and these results may provide important information for biodiversity management in cities.

## 1 INTRODUCTION

An interesting problem in ecology and conservation biology is determining the survival mechanisms of plant or animal populations in fragmented landscapes (Fahrig, 2003). A common assertion is that gene flow among populations (Ellstrand & Elam, 1993; Young, Boyle, & Brown, 1996) and long‐distance dispersal of seeds (Bohrer, Nathan, & Volis, 2005; Cain, Milligan, & Strand, 2000) could play an important role in species viability, population survival, and structure especially in human‐fragmented populations. For these reasons, corridors (e.g., urban greenways) have become important features of biodiversity management (Damschen, Haddad, Orrock, Tewksbury, & Levey, 2006).

An urban environment is particularly characterized by a high level of habitat fragmentation (Schmidt, Poppendieck, & Jensen, 2014). According to Rebele (1994), urban ecosystems present special features, such as mosaic phenomena, species invasion, and extinction processes, where specific perturbation regimes can influence the dynamics and structure of plant and animal populations.

To preserve biodiversity, cities are interested in creating corridors between green areas to enable flora and fauna to move and survive throughout their territory. Furthermore, constructing buildings and maintaining efficient corridors simultaneously require an understanding of how species can spread across urban spaces. Indeed, all species do not have the same dispersal behavior (e.g., Mörtberg & Wallentinus, 2000; Vergnes, Le Viol, & Clergeau, 2012). Moreover, preserving biodiversity and abundance in cities often conflicts with keeping clean public spaces, such as streets and pavements (Dempsey & Burton, 2012). Better understanding the role of green urban spaces and corridors in the dynamics of plants with different biological characteristics could help designing accurate technical procedures, like minimization of targeted species removal.

Metapopulation theories are used to help describe and understand the species dynamics in fragmented landscapes (Moilanen, 2004; Perry & Gonzalez‐Andujar, 1993). Plant species may seem specifically suitable for metapopulation analysis thanks to their immobility, restricted dispersal, and strong spatial structure (Husband & Barrett, 1996). The theories suggest that species survive in patchy landscapes via an equilibrium between local colonization and extinction (Dornier, Pons, & Cheptou, 2011; Levins, 1969). Many metapopulation models have been described and studied, and two families of models in particular are assumed to fit species dynamics in patchy landscapes, such as urban ones (Breuste, Niemelä, & Snep, 2008). The Levins model (LM; Levins, 1969) assumes that a species moves from one place to another in a stepwise fashion governed by its dispersal capacity and potential habitat distribution. In the LM, the probability that a patch will be colonized is linked to the distance to adjacent occupied patches. The propagule rain model (PRM; Gotelli, 1991) assumes that the species are mostly located outside the system in large source populations that send large numbers of propagules to every patch with the same probability; that is, the probability of colonization is independent of the species occurrence in neighboring patches. In the LM and PRM, the probability of local extinction is fixed for any distance to the next occupied patches. Hanski (1982) incorporated a “rescue effect” (Brown & Kodric‐Brown, 1977) into the LM (LM + R), which reduces the local extinction probability based on the supply of seeds from neighboring occupied patches. A rescue effect may also be added to the PRM (PRM + R). These four metapopulation models can represent the species dynamics depending on whether the colonization and extinction probabilities are governed by local occurrences or not (Gotelli, 1991).

At the street level, sets of urban trees can be considered as metapopulations for wild plants growing at their bases. Indeed, in such urban situations, plant species live in spatially distinct inhabited patches within an inhospitable matrix (Pellegrini & Baudry, 2014; Schmidt et al., 2014). Thanks to data of survey over years, we can calculate colonization and extinction probabilities based on species occupancy for each species yearly. Simulating metapopulations can help us to understand whether species move according to stepwise models or seed rain models.

This paper reports the results of a study on the population dynamics of plants growing at the bases of trees aligned along streets (hereafter called “patches”) in a highly urbanized district. We considered tree bases, that is, squares or circles of soil around tree trunks, at the bottom of alignment trees, as favorable patches for herbaceous plant species growth. In Paris (France), nearly 100,350 trees planted on sidewalks could serve the ecological function of habitats and/or corridors between city parks and gardens (Vergnes et al., 2012).

**Figure 1:**
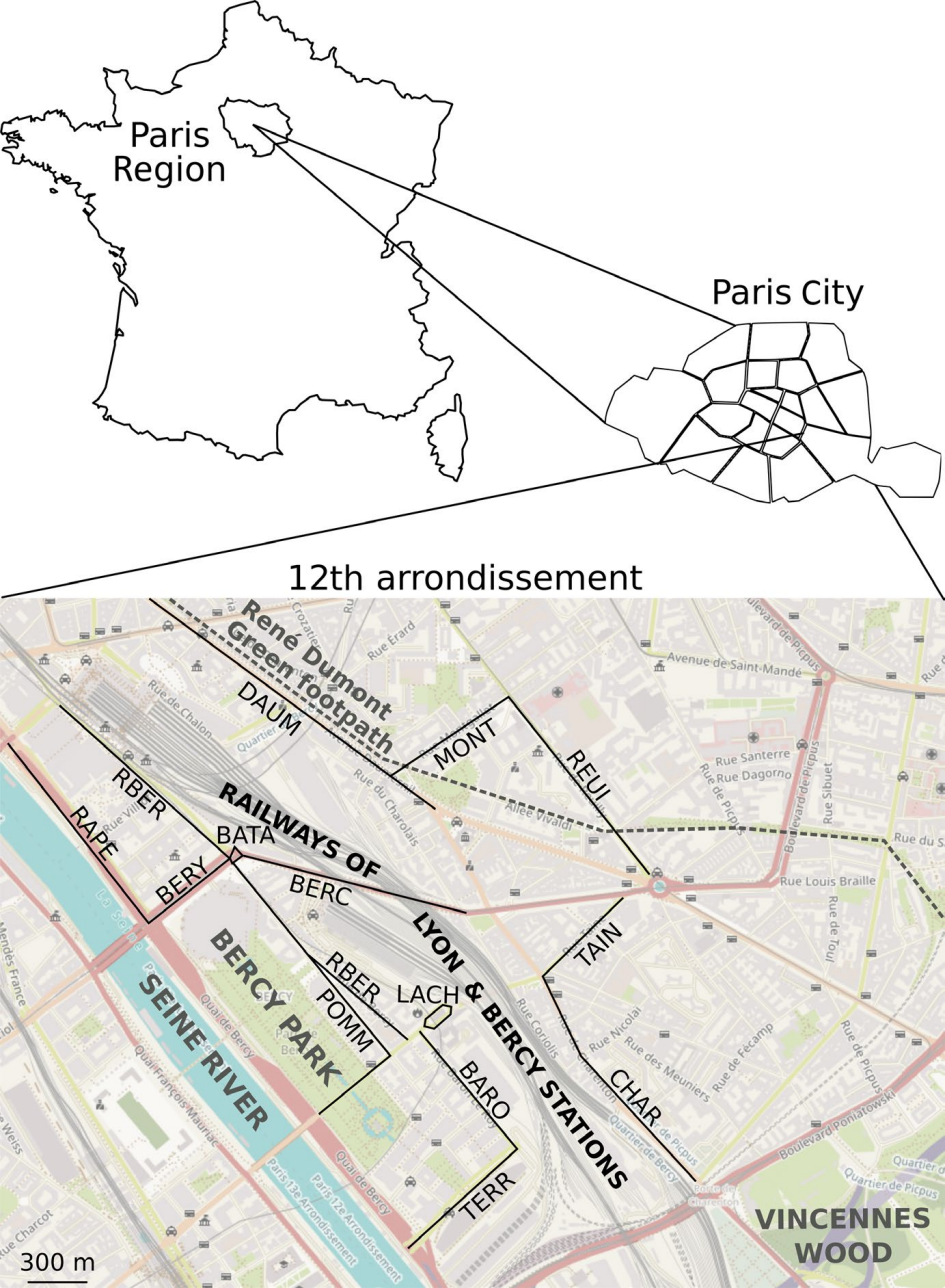
Map of the Bercy District in the 12th arrondissement of Paris (France) where the floristic inventories were performed. The names of the 15 streets, represented by black lines, are given in Table 1

In 2012, Dornier & Cheptou studied the colonization/extinction dynamics of the annual plant *Crepis sancta* (L.) Bornm. (Asteraceae) growing at urban tree bases in Montpellier (France). These authors showed that one part of the network was best described by the PRM, whereas the other part was best described by Levins’ metapopulation dynamics (LM), and they assumed that an external source of propagules occurred in the first part of the network. In this study, we used the same concept of a stochastic patch occupancy model (SPOM) to assess the nature of the population dynamics for species growing at the bases of trees aligned along streets in the Bercy District of Paris (France; Figure 1). We chose this district because of its high number of urban trees and location near the Seine River. Furthermore, this district includes a large park (Bercy Park), a green footpath (René Dumont footpath), and the railways of the Lyon and Bercy stations, which are all potential sources of certain plant populations in the surrounding streets. SPOMSIM software (Moilanen, 2004) yielded models that best fit the annual data (collected from 2009 to 2015) on the presence/absence of 15 species in each of the 1,324 tree bases distributed over 15 district streets. In addition, the results of the models were compared among (a) species with different biological characteristics; and (b) streets at different distance to possible external seed sources, that is, the green spaces of the district.

This study was performed to identify the plant species dynamics in a highly urbanized district. The goals were to (a) determine theoretical metapopulation models followed by each species; and (b) link these results to biological traits of the species and the spatial arrangement of the district. Our study aimed to assess the accuracy of tree bases as corridors to improve knowledge in urban ecology on the dynamics of plant species. Furthermore, this work should provide information to managers of urban spaces to allow them to take appropriate management decisions.

## 2 MATERIALS AND METHODS

### 2.1 Study area and geographic characteristics

The study was performed in the 12th administrative district of Paris (France; 48°50′26.91″N, 2°23′17.46″E; Figure 1), which covers an area of approximately 6.4 km^2^ and contains 26 streets primarily lined by buildings that may have small gardens, railways leading to the Bercy and Lyon stations, a large public garden (Bercy Park), and the green René Dumont footpath. The study area lies on the north side of the Seine River and the west side of the Vincennes Wood and harbors a human population of ca. 144,000 inhabitants, which is equivalent to ca. 23,000 inhabitants/km2 (INSEE, 2017).

### 2.2 District characteristics

Of the 26 streets in the district, only the 15 streets that included more than 30 tree bases were retained for analysis, thus resulting in an annual inventory of 1,324 tree bases. To examine the effect of green spaces as potential seed sources, we calculated the Euclidean distances between all patches in the studied streets and each of the district green spaces (i.e., the Seine River, Bercy Park, René Dumont footpath, and Lyon and Bercy railways; Figure 1) using the geographic information system software ArcGIS 10.2 (ESRI, 2012). Then, we calculated the mean of the Euclidean distances from all patches in each street to each green space and chose the smallest mean distance value of each street to the nearest green space (Table 1). Street length, width, and area were also measured with ArcGIS 10.2 (ESRI, 2012). We also studied the possible effect of the street orientation, deduced from district satellite image, according to the airflow following the Seine River to see if it was parallel (i.e., subjected to the airflow) or perpendicular (i.e., protected by buildings bordering the streets) (Omar, Al Sayed, Barré, Halwani, & Machon, 2018). Note that there is no significant difference in pedestrian frequency or the quantity of traffic between parallel versus perpendicular streets (Fisher tests, not shown).

**Table 1:** Features of the 15 streets for which the wild flora population dynamics was modeled and results of SPOMSIM modeling

Street name	Street features				Percentage of each model^a^		
	Abbreviation	Number of tree bases	Smallest distance^b^ (m)	Nearest green space	LM	LM + R	PRM
Rue Baron le Roy	BARO	62	166	Railways of Lyon and Bercy stations	53	13	33
Place du Bataillon du Pacifique	BATA	31	63	Railways of the Lyon and Bercy stations	40	0	60
Boulevard de Bercy (1)	BERC	126	33	Railways of the Lyon and Bercy stations	47	27	27
Boulevard de Bercy (2)	BERY	99	148	Seine River	40	13	47
Rue de Charenton	CHAR	144	41	Railways of Lyon and Bercy stations	53	13	33
Rue Daumesnil	DAUM	186	7	René Dumont footpath	27	7	67
Rue Joseph Kessel	KESS	69	3	Bercy Park	33	13	53
Place Lachambeaudie	LACH	31	13	Railways of Lyon and Bercy stations	27	0	73
Rue Montgallet	MONT	52	125	René Dumont footpath	40	13	47
Rue Pommard	POMM	39	17	Bercy Park	27	0	73
Quai de la Rapée	RAPE	97	64	Seine River	47	13	40
Rue de Bercy	RBER	136	88	Railways of the Lyon and Bercy stations	40	7	53
Rue de Reuilly	REUI	145	224	René Dumont footpath	60	7	33
Rue Taine	TAIN	62	38	Railways of the Lyon and Bercy stations	20	7	73
Rue des Terroirs de France	TERR	45	177	Seine River	47	7	47

Notes

The street names and their abbreviations, number of tree bases per street.

a The percentage of species dynamics that conformed to each model in each street are provided.

b The smallest Euclidean distance between each street and the closest green space (m) to the nearest green space.

### 2.3 Floristic inventories

We monitored 1,324 tree bases distributed along 15 streets or avenues in the district (Figure 1, Table 1). All patches were localized with a GPS (Global Positioning System), and their state (occupied or empty) was noted to allow population turnover estimates. A list of all wild vascular plant taxa was established once a year in each patch during May or June for 7 years from 2009 to 2015 except in 2013 because of a lack of observers. Species were identified based on French flora (Tison & de Foucault, 2014), and the taxonomic reference is the French Flora Reference TAXREF v8.0 (Gargominy et al., 2016).

### 2.4 Species biological characteristics

For this study, we only considered species that were present in at least 200 patches in a survey to facilitate running the models. For each of the resulting 15 identified species, 100 dry seeds were weighed and examined to determine whether they had a dispersal system (e.g., wings, pappus). Seed longevity in a soil bank was deduced from Thompson, Bakker, and Bekker (1997), and species persistence was classified as short‐term (<5 years) or long‐term (>5 years) according to the seed persistence in the soil. We also determined the species’ life span and maximum height using the database provided by the collaborative network of French botanists “Tela Botanica” (http://www.tela-botanica.org; Table 2).

**Table 2:** Features of the 15 species for which population dynamics was modeled and SPOMSIM modeling results

Species	Biological features					Percentage of each model^a^		
	Seed weight (mg)	Seed dispersal system^b^	Seed longevity category^c^	Species’ life span	Maximum plant height (cm)	LM	LM + R	PRM
*Capsella bursa‐pastoris*	2.5	0	2	Annual	50	53	13	33
*Chenopodium album*	71.4	0	2	Annual	100	53	0	47
*Conyza canadensis*	15	0	1	Annual	80	47	7	47
*Hordeum murinum*	366.4	1	1	Annual	50	40	20	40
*Lactuca serriola*	45	1	1	Annual	100	40	7	53
*Lolium perenne*	228	0	1	Perennial	60	47	13	40
*Plantago major*	250	1	2	Perennial	50	20	0	80
*Poa annua*	30	0	2	Annual	30	33	13	53
*Polygonum aviculare*	92.1	0	2	Annual	80	33	7	60
*Senecio inaequidens*	30	0	2	Perennial	80	33	7	60
*Senecio vulgaris*	20	1	1	Annual	60	53	0	47
*Sisymbrium irio*	7.3	0	1	Annual	90	47	13	40
*Sonchus oleraceus*	24.7	1	2	Annual	80	27	0	73
*Stellaria media*	35	0	1	Perennial	30	33	33	33
*Taraxacum campylodes*	50	1	2	Perennial	40	33	13	53

a Percentage of streets in which each species’ dynamics conformed to each model.

b Presence of a seed dispersal system: 1; no dispersal system: 0.

c Short‐term persistent seeds: 1; seeds with long‐term persistence: 2; maximum plant height.

### 2.5 Model definitions

To test the metapopulation dynamics of each species in each street, we constructed four different scenarios based on the dependence of colonization and extinction probabilities on occupancy patterns. Different metapopulation models were compared using the software SPOMSIM, which models the presence/absence of a species in inhabited patches as a Markov chain (Moilanen, 1999, 2000, 2002, 2004; Ovaskainen & Hanski, 2004). We considered each species in each street a distinct metapopulation and each tree base a spatially referenced patch. In our models, we assumed that the patch area and the distances between two successive patches were constant throughout the district, which is true in the first approximation.

In each year *t*, an empty patch *i* might be colonized with probability *C_i_*(*t*) while an occupied patch might undergo extinction with probability *E_i_*(*t*).

#### 2.5.1 Colonization probability

The PRM (Gotelli, 1991), in which colonizing propagules are derived from large and frequently unknown populations, assumes that the colonization probability is constant, meaning that it is not affected by the distance to the occupied patches in the metapopulation, which could provide an indication of external colonization (Dornier et al., 2011). Thus, we have the following:

(1) $$C_{i}\left(t\right)=C_{i},$$

where *C_i_* is the intrinsic colonization rate of patch *i*.

However, the LM (Levins, 1969) assumes that the colonization probability depends on the distance to the nearest occupied patches. SPOMSIM software defines the distribution of the dispersal distances (or the dispersal kernel) of each species in each street as a negative exponential (according to Hanski, 1994; Kot, Lewis, & van den Driessche, 1996; Okubo, 1980; Shaw, 1994, 1995) as cited in Moilanen (2004):

(2) $$D\left(d_{\mathrm{ij}},\alpha \right)=\text{exp}\left(-\alpha d_{\mathrm{ij}}\right),$$

where *d_ij_* is the distance between patches *i* and *j*, and parameter *α* defines the distribution of the dispersal distances (1/*α* is the average dispersal distance and *αd_ij_* can be interpreted as the mean number of colonized patches; Hanski, 1994).

To define the colonization probability, SPOMSIM uses the connectivity function *S_i_*(*t*) (Moilanen & Nieminen, 2002), which describes the state of occupancy of the metapopulation:

(3) $$S_{i}\left(t\right)=\Sigma_{i}\neq j\;O_{i}\left(t\right)D\left(d_{\mathrm{ij}},\alpha \right),$$

where *O_i_*(*t*) = 1 for occupied and *O_i_*(*t*) = 0 for empty patches, and *D*(*d_ij_*,*α*) is the previously defined dispersal kernel.

Colonization is a function of connectivity, and it is defined as follows:

(4) $$C_{i}\left(t\right)=1-\text{exp}\left(-yS_{i}\left(t\right)\right)=1-\text{exp}\left(-y\Sigma i\neq j\;O_{i}\left(t\right)D\left(d_{\mathrm{ij}},\alpha \right)\right),$$

where *y* is a parameter depending on each street.

#### 2.5.2 Extinction probability

Symmetrically, extinction might be fixed or dependent on the presence of nearby occupied patches (Hanski & Ovaskainen, 2000; Ovaskainen & Hanski, 2002, 2004). In the first case, that is, in LM and PRM, the extinction probability is equal to the probability that patch *i* has not been colonized multiplied by *E_i_*, which is the intrinsic rate of the extinction of each species:

(5) $$E_{i}\left(t\right)=E_{i}\left(1-C_{i}\left(t\right)\right).$$

In the second scenario, the contribution of neighboring occupied patches to the colonization probability of empty patches is affected by a “rescue effect.” Thus, because of past migration, a population is saved from extinction by its large population size (Brown & Kodric‐Brown, 1977; Hanski, Moilanen, & Gyllenberg, 1996), which lowers the extinction probability when *R* tends toward 0:

(6) $$E_{i}\left(t\right)=E_{i}{\left(1-Ci\left(t\right)\right)}^{R},$$

where parameter *R *determines the strength of the rescue effect. Levins' and PRMs with rescue effect are noted LM + R and PRM + R, respectively.

### 2.6 Model selection and data analysis

#### 2.6.1 Metapopulation model selection

We estimated the best metapopulation model for each of the 15 species in each of the 15 streets, using the method developed by Moilanen (1999) implemented by the SPOMSIM software (Moilanen, 2004), which resulted in 225 models (Appendix A). The software estimated the parameters of each metapopulation model for each street–species combination and calculated an Akaike information criterion corrected (AICc for small samples) for each model (Moilanen, 2004). Thus, for each street–species combination, the model (i.e., LM, PRM, LM + R, or PRM + R) that fitted best the data was the one with the smallest AICc (AICc for the best selected model is shown in Appendix A).

#### 2.6.2 Data analysis

We then calculated the proportion of species in each street that conformed to each model (Table 1) and the proportion of streets in which each species conformed to each model (Table 2). We used generalized linear models (GLMs, R package MASS) to test correlations between proportions of metapopulation models (as response variables) and species biological characteristics or geographic features of the district (i.e., street distance to the nearest green space, length, width, area, and orientation to the predominant winds created by the Seine River) with a quasi‐binomial error distribution (Venables & Ripley, 2002; Zuur, Ieno, Walker, Saveliev, & Smith, 2009).

At species level, we tested the potential relationship between the proportion of streets that conformed to PRM and the species’ biological traits (seed weight, seed dispersal system, seed longevity in the soil bank, and species’ life span). We also studied the relationship between the proportion of streets that conformed to the models with rescue effect (LM + R or PRM + R) and the maximum plant height. At street scale, we tested the relationship between the proportion of PRM, LM + R, and PRM + R per street and the geographic features of the district (i.e., street length, width, area, orientation relative to the airflow created by the Seine River, and distance to the nearest green space).

We then used the variance inflation factor (VIF) function in the R platform (Fox et al., 2014) to discard possible variables that generated excessive collinearity with the other variables in the full models. All variables showed a VIF value <2, meaning that considerable evidence of multicollinearity was not observed (Chatterjee & Hadi, 2015)

Each explanatory variable was assessed via a visual inspection of the plot to determine whether a quadratic transformation of the variables (i.e., a nonlinear relationship) was required in the GLM using a generalized additive model (GAM) with the R package mgcv (Wood, 2017). Quadratic effects were not observed among our variables. Spatial autocorrelations were assessed among the model residuals with a Mantel test, and we found a nonsignificant spatial autocorrelation among the model residuals for the proportion of the PRMs by street. Thus, we assumed that spatial autocorrelations were absent or negligible. We also examined the relative variance explained by calculating an adjusted *R*‐squared value for the models.

Obvious overdispersion issues were not detected in models for the proportion of the PRMs and models with the rescue effect based on the species because all values fell between 0.8 and 1.25. We validated the models by checking the residual plots. The observed residuals were consistent with the stochastic errors. We used the allEffects function (R package Effect) to obtain the predicted values from the GLMs and the ggplot function to build the figures presented in the Results section (Wickham, 2010; Wilkinson, 2005). All statistical analyses were performed using R version 3.4.3.

## 3 RESULTS

### 3.1 Modelized species and streets

A total of 225 street–species combinations (15 streets and 15 species) were successfully modeled by SPOMSIM (Tables 1 and 2; see Appendix A for the complete list of the surveyed species). We defined the best metapopulation model for each street–species combination. Overall, the results were 50% of PRM, 40% of LM, and 10% of Levins' model with rescue effect (LM + R). The PRM with rescue effect (PRM + R) was never found; that is, no species × street pair conformed to this model.

### 3.2 At species level: biological drivers of the PRM proportions

**Figure 2:**
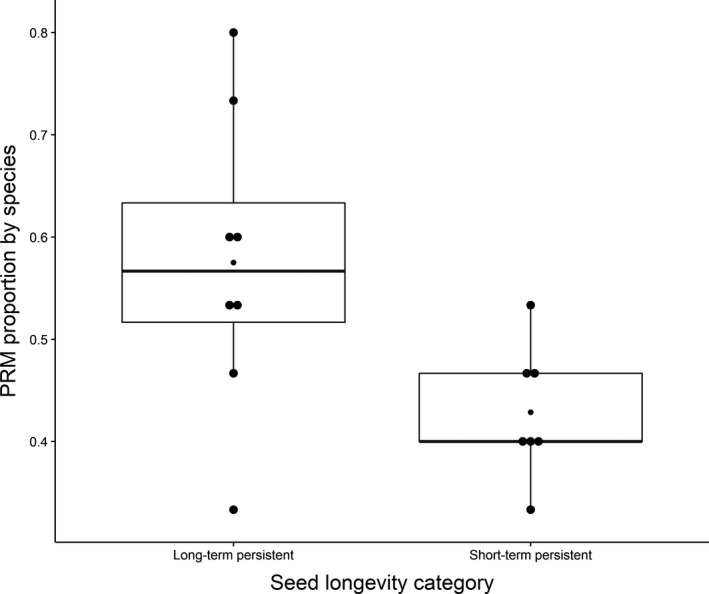
Relationship between seed longevity and propagule rain model (PRM) proportion by species (long‐term persistent species: PRM mean = 0.57; short‐term persistent species: PRM mean = 0.43; *p*‐value = 0.017; *R* ^2^ = 0.53)

We tested the correlations between PRM proportion by species, the three seed characteristics, and species’ life span. Contrary to our expectation, we observed no difference in PRM proportions between perennial and annual species (*p*‐value = 0.694). Our results also indicated that PRM proportion was not correlated with seed weight (*p*‐value = 0.965) or presence/absence of a seed or fruit dispersal system (*p*‐value = 0.066). Nevertheless, a strong relationship was observed between seed longevity in soil bank and PRM proportion (Figure 2; *p*‐value = 0.027). Indeed, species with seeds that present long‐term persistence (i.e., more than 5 years of persistence in the soil bank; *Capsella bursa‐pastoris, Chenopodium album, Plantago major, Poa annua, Polygonum aviculare, Senecio inaequidens, Sonchus oleraceus*, and *Taraxacum campylodes*; PRM mean = 0.57) had a higher PRM proportion than seeds with short‐term persistence (i.e., <5 years of persistence in the soil bank; *Conyza canadensis*, *Hordeum murinum, Lactuca serriola, Lolium perenne, Senecio vulgaris, Sisymbrium irio*, and *Stellaria media*; PRM mean = 0.43; *p*‐value = 0.017 and *R* ^2^ = 0.53; Figure 2).

### 3.3 PRM proportion by street and distance to the nearest green space

**Figure 3:**
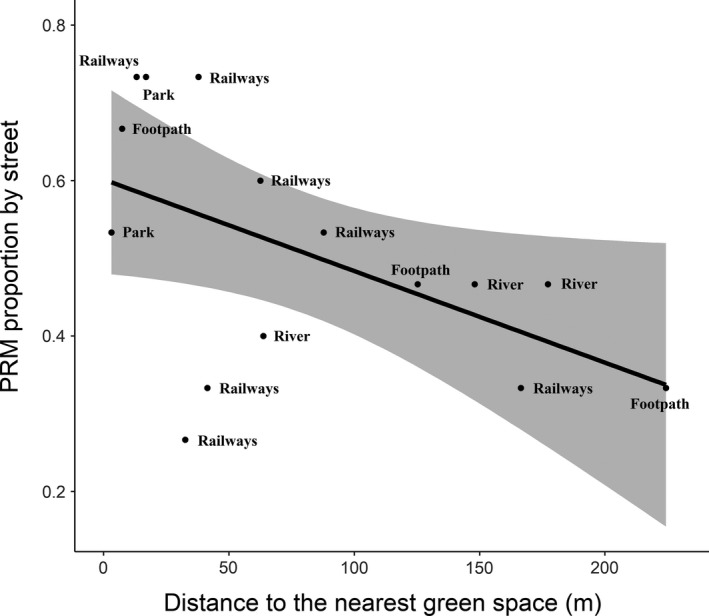
Relationship between mean street distance to the nearest green space (in m) and propagule rain model (PRM) proportion by street. PRM proportion by street decreases with street distance to the nearest green space (*b* = −0.0048; *p*‐value = 0.048; *R* ^2^ = 0.27). Types of the nearest green space are indicated on the figure for each dot (=street). Black line represents predicted values of the proportion of PRM by street from the generalized linear models, and gray zone shows the associated 95% confidence interval

Globally, the proportion of PRM by street decreased with the mean street distance to the nearest green space (slope of the regression *b* = −0.0048; *p*‐value = 0.048; *R* ^2^ = 0.27; Figure 3).

According to our results, the effect of the street characteristics (length, width, area, and orientation relative to the airflow created by the Seine River) was not significant on the proportion of PRM by street (results not shown).

### 3.4 PRM proportion by species

**Figure 4:**
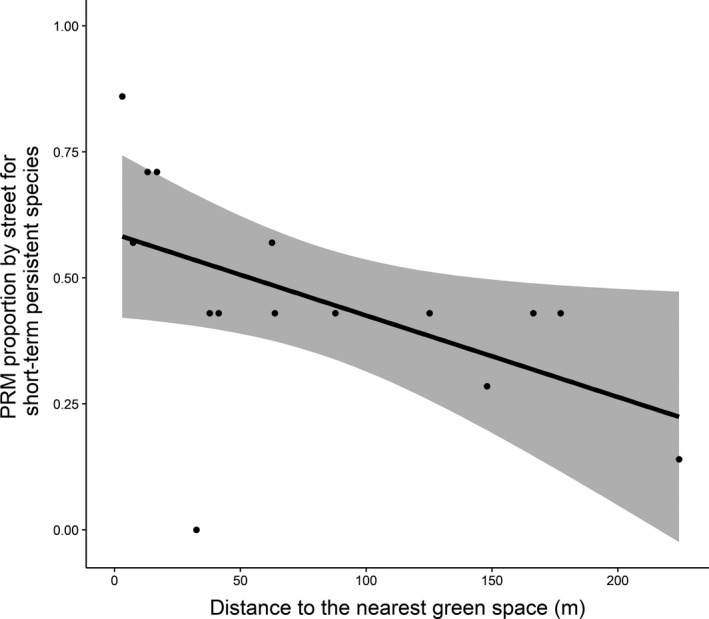
Relationship between propagule rain model (PRM) proportion by street and mean distance to the nearest green space (in m) for short‐term persistent species only. PRM proportion for short‐term persistent species decreases with distance to the nearest green space (*b* = −0.0069; *p*‐value = 0.049; *R* ^2 ^= 0.24). Black line represents predicted values from the generalized linear models, and gray zone shows the associated 95% confidence interval

A strong relationship was observed between PRM proportion by species and seed bank type and between PRM proportion by street and distance to the nearest green space. Therefore, we tested separately the relationship between PRM proportion by street and distance to the next green space for species in each seed bank category. Our results were significant for seeds with short‐term persistence (*b* = −0.0069; *p*‐value = 0.049; *R* ^2^ = 0.24; Figure 4) but not for seeds with long‐term persistence (results not shown).

### 3.5 Rescue effect proportion by species or street

**Figure 5:**
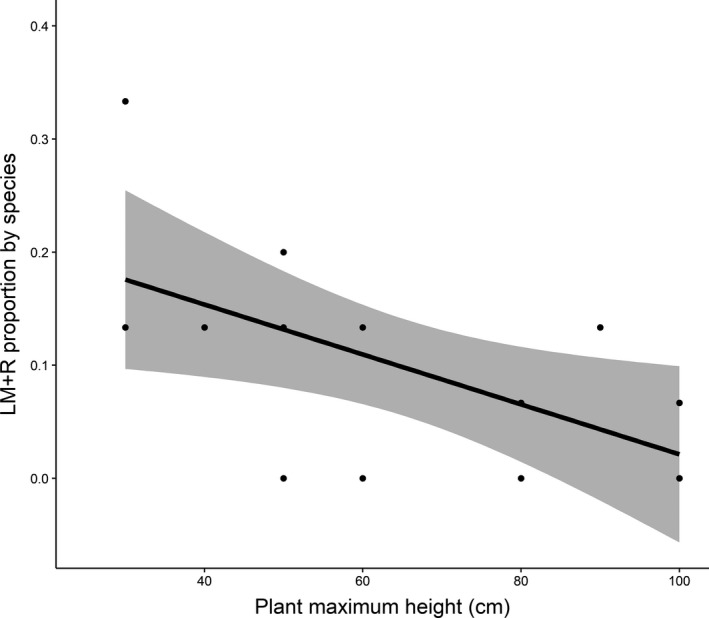
Relationship between proportion of Levins’ model with rescue effect (LM + R) by species and maximum plant height (cm). Shorter species' dynamics more frequently conformed to LM + R model compared to taller species (*b* = −0.026; *p*‐value = 0.021; *R* ^2^ = 0.3). Black line represents the predicted values from the generalized linear models, and gray zone shows the associated 95% confidence interval

Only 10% of the models included a rescue effect, and all were LM + R and not PRM + R. When analyzing the biological features of species whose dynamics conformed to LM + R model, the rescue effect appeared to be correlated with species maximum height (*b* = −0.026; *p*‐value = 0.021; *R* ^2 ^= 0.30; Figure 5). Street characteristics (length, width, area, orientation relative to the predominant winds created by the Seine River, or proximity to a green space) were not correlated with rescue effect proportion (results not shown).

## 4 DISCUSSION

Data from the intensive inventories performed yearly between May and June from 2009 to 2015 were used to model the colonization and extinction dynamics of 15 species growing at the bases of 1,324 urban trees in 15 streets in the Bercy District; however, data for 2013 were not included. Nevertheless, a verification in removing randomly one other year of data for the species *T. campylodes* resulted in the same best‐fit model for species–street combination (result not shown).

### 4.1 Propagule rain model

More than 50% of the metapopulations of *L. serriola*, *P. major*, *P. annua*, *P. aviculare*, *S. inaequidens*, *S. oleraceus*, and *T. campylodes* species conformed to PRM defined by Gotelli (1991). This likely indicates that an external seed source is feeding the system with large quantities of seed; therefore, the possible movement of species among tree bases is negligible. Similar results were observed with genetic tools on *S. inaequidens* in Paris (Blanchet et al., 2015).

We assumed that species with seeds equipped to be transported by wind, epizoochory (i.e., in fur or feathers of animals or attached to human clothes), or endozoochory (i.e., via ingestion by birds or mammals; Corlett, 1998; Hurka & Haase, 1982) as well as those with light seeds that could be carried by wind could preferentially escape from green spaces. However, no correlation was observed between proportion of PRM and seed mobility based on dispersal system or seed weight. This absence of correlation is consistent with the results obtained formerly by Omar et al. (2018) on the aggregated and/or spread distribution of plant species in the district of Bercy.

Nevertheless, according to Sukopp (2004), all seed types produced by herbaceous species in tree bases (with or without a dispersal system) are rather small and might be easily dispersed by human activity, for example, transported in mud stuck to shoes or car tires (Von Der Lippe & Kowarik, 2007). Actually, Bercy Park and René Dumont footpath are crossed everyday by many pedestrians and cyclists who potentially contribute to bring seeds into the streets of the district. In wasteland railways, which are not accessible to pedestrians, many other vectors could introduce the seeds in the streets. Among them, crosswind effects induced by train's slipstream (Barcala & Meseguer, 2007) or animals (like birds, dogs, or rats), but without favoring any type of seed on their dispersal device or weight.

Our model took also into account other geographic parameter which could impact population dynamics, like the street orientation to the predominant winds in the direction of the Seine River. According to Omar et al. (2018), the airflow induced by the river could transport a number of different species in its direction into the streets of the studied district. Thus, the seeds could use the lanes formed by the buildings lining the parallel streets but could be stopped by the buildings bordering the perpendicular streets. In fact, the buildings could have a windbreak function, reducing crosswind effects and thus yielding a good seed retention system (Damschen et al., 2006; Omar et al., 2018). Nevertheless, our results on the street orientation to the predominant winds created by the Seine River were not correlated with the proportion of PRM by street.

No significant difference was found between mean PRM proportion of annual and perennial plants, but the results showed that species that mainly conformed to the PRM were those that can constitute a long‐term seed bank in the soil (Figure 2). Thus, we can assume that this type of PRM dynamics for long‐term persistent species could be primarily caused by the emergence of seedlings from a soil seed bank accumulated over a long period rather than from clouds of seeds in the vicinity of green spaces, as discussed by Dostál (2005) and Kalamees and Zobel (2002). In 1997, Kalisz, Horth, and McPeek explained the ecological role of dormancy in highly fragmented landscapes for species whose regional persistence relies on metapopulation function (e.g., *Collinsia verna*); dormancy may allow some populations to be restored. According to these authors, the fitness value of seed banks helps the increase of plant populations that are experiencing habitat degradation, fragmentation, and/or isolation.

Nevertheless, streets hosting the largest number of species conformed to PRM were mostly situated in a 100‐m range from a green space (Figure 1; Table 1), and PRM was the best model for short‐term persistent seed species' dynamics in the neighborhood of green spaces (Figure 4). For these reasons, we assumed that green spaces could still play a strong role in the colonization of tree bases, especially for short‐term persistent seed species. For example, the two nearest streets to Bercy Park (Joseph Kessel and Pommard streets, which are following the park; Figure 1) show high proportions of PRM (53% and 73%, respectively). The potential seed source role of Lyon and Bercy train stations seems less obvious for certain adjacent streets, like Bercy street. Moreover, even if the green sides of railways host quantities of wild plants, these areas do not seem to provide seeds to all neighborhood streets. For example, Lachambeaudie and Taine streets are very close to the railways and both show 73% of PRM, whereas Charenton and Bercy streets show respectively 33% and 27% of PRM. However, Charenton street is separated from the railways by a 2.5‐m‐high wall and Bercy street is passing under the railway bridge, which could explain the lack of seed propagation (Figure 1). This inhibition could explain the low percentage of species whose dynamics conformed to a PRM in these streets (Table 1).

Our way to delimitate what we considered as metapopulation has certainly also an impact on which model the species conformed to. When streets were taken as independent sampling units (the limits of each metapopulation), the effect of adjacent streets was not taken into account and the effect of the distance between occupied patches was underestimated. That means that the "unknown" source that kept colonization probability independent of the occupied patches within metapopulation could also be patches from adjacent streets.

### 4.2 Levins’ model

Five species’ populations (*C. bursa‐pastoris*, *C. album*, *L. perenne*, *S. vulgaris*, and *S. irio*) frequently conformed to LM. As previously mentioned, the ability of certain species to constitute a seed bank in the soil could disturb the simple stepping‐stone function adopted by certain species, resulting in a smaller proportion of LM. The nondetectable seed bank, which allows plants to emerge after several years of absence, implies false extinction and recolonization which biases toward an overestimation of PRM and underestimation of LM dynamics (Eriksson, 1996; Freckleton & Watkinson, 2002; Pluntz et al., 2018). This could explain that LM dynamics was more frequently encountered for short‐term persistent seed species. Nevertheless, when we focused only on short‐term persistent seed species, the same pattern of distribution of PRM versus LM in the neighborhood of the green spaces was observed. This result confirmed the potential impact of green spaces on the dynamics of the species in the streets.

Six streets (Baron le Roy, Bercy, Charenton, Rapée, Reuilly, and Terroirs de France) hosted a high proportion of species whose dynamics followed LM (47% or more). The Reuilly street, which showed the highest proportion of LM (60%), is also the farthest from a green space. Other streets, like Charenton and Bercy, are obviously isolated from seed sources by buildings. Thus, we can suppose that without any tree bases in these streets, the species following LM would not be able to enter some districts and reach spaces embedded in the dense urban matrix. In such cases, tree bases appear to play an essential role as corridors for species with short seed longevity and in streets that are bordered by high buildings.

### 4.3 Models with rescue effect

In the studied district, the PRM metapopulations did not appear to benefit from a rescue effect for extinction processes: We found no PRM + R models. Therefore, in all cases, fixed extinction models were the best fit for the species.

Some of the species that conformed to LM presented reduced extinction by the presence of occupied neighboring patches (rescue effect, e.g., *H. murinum*, *S. media*). Perennial species versus annual ones did not conform to model with rescue effect. At the street scale, none of the particular street characteristics (street length, width, area, orientation to the predominant winds in the direction of the Seine River, and distance to nearest green space) were correlated with proportion of LM with rescue effect. However, at species level, our analysis showed that the highest proportion of species that conformed to the LM with rescue effect were those that had the smallest size (Figure 5).

We assume that weeding in the streets could explain the observed results. The regular clearing of vegetation via hoes or brush cutters occurs once a year in the streets of this district, according to the technical services in charge of cleaning Paris streets. This weeding could regularly cause the extinction of a certain number of plant populations. Because long‐lived seed species can remain as seeds in the soil, the most threatened species by weeding could be the ones with short‐lived seeds, even if they are abundant. The results also show that certain species are not similarly managed, with taller plants appearing to be more completely eradicated while shorter species appearing to be retained to a greater degree. Indeed, *C. album*, *C. canadensis, L. serriola, P. aviculare, S. inaequidens, S. irio*, and *S. oleraceus*, which present heights that can reach more than 80 cm, had a LM with rescue effect percentage ≤7%. However, *C. bursa‐pastoris*, *P. annua, S. media*, and *T. campylodes*, which grow up to 50 cm, had a LM with rescue effect percentage >13%. The most important LM with rescue effect proportion is for *H. murinum* despite its high size. Nevertheless, this species has a very strong capacity to disperse its seeds by sticking to the fur of passing animals or the clothing of people and thus recolonize tree bases after eradication.

## 5 CONCLUSION

In conclusion, our study showed that SPOMs can provide important insights into plant local dynamics. Because this type of software cannot simulate a seed bank, the results are biased toward an overestimation of PRM. Nevertheless, when a seed bank occurs, extinction and colonization are always difficult to model (Eriksson, 1996; Freckleton & Watkinson, 2002). However, this study showed that street species could originate from green spaces and invade surrounding streets through a seed rain process. Some urban elements like walls or bridges could constitute barriers for species dissemination. Species that can form a seed bank accumulate at tree bases and finally disperse throughout the district. Other more ephemeral species colonize different areas by moving stepwise from one tree base to the others. This result is consistent with the observations described in Bossuyt and Hermy (2003), who determined the potential role of persistent soil seed banks in restoring plant communities. The species with long‐term persistent seeds are often ruderal or competitive species, typical of disturbed sites. They can survive for a long period in the soil and colonize the newly established community by seed dispersal or by germination of seeds buried in the soil bank. They could thus be dispersed more extensively throughout the district. The extinction process, even if not perfectly considered because a seed bank was not included in the model, convincingly showed the effect of weeding performed by the city, which spared the shortest species while regularly eradicating the taller ones.

City managers should consider tree bases to act as corridors among biodiversity kernels (Newmark, 1996), which link urban green spaces separated by considerable distances. Thus, tree bases should be considered for the preservation of urban biodiversity. In planting more alignment trees at sidewalks, which are fed by the seeds from these parks, and in protecting them from intense practices and human destruction, better conditions would be provided for spontaneous wildlife and other ecological functions would be favored. This spatial planning strategy should be adopted to provide the best habitats and conditions for species dynamics in compacted cities. These actionable activities are important because quality of biodiversity influences well‐being of citizens (Tzoulas et al., 2007) and participates in other ecosystem services like air filtration (Weber, Kowarik, & Säumel, 2014) or microclimate regulation (Bolund & Hunhammar, 1999).

## CONFLICT OF INTEREST

None declared.

## AUTHOR CONTRIBUTIONS

Mona OMAR and Laure SCHNEIDER‐MANOURY led the writing of the manuscript. Mona OMAR analyzed and interpreted the data. Kévin BARRÉ and Nazir AL SAYED contributed to the statistical analyses. Nathalie MACHON conceived the ideas, designed methodology, contributed to the writing, and supervised the research group. Jalal HALWANI revised the manuscript for important intellectual content. All authors contributed critically to the drafts and gave final approval for publication.

## Data Availability

Data used for the analysis is uploaded in a Dryad repository (https://doi.org/10.5061/dryad.61sr186).

## APPENDIX A

The best metapopulation model for each of the 15 species in each of the 15 streets followed by each species and street determined using the Akaike information criterion corrected for small samples estimated by SPOMSIM software (Moilanen, 2004). The parameter *α *defines the distribution of dispersal distances (1/*α *is the average dispersal distance). The street names and their abbreviations are given in Table 1.

### SPECIES AND MODEL ABBREVIATIONS

*Po.an: Poa annua; La.se: Lactuca serriola; Pl.ma: Plantago major; Si.ir: Sisymbrium irio; Ch.al: Chenopodium album; Lo.pe: Lolium perenne; Se.vu: Senecio vulgaris; Ta.ca: Taraxacum campylodes; Ho.mu: Hordeum murinum; ca.bu: Capsella bursa‐pastoris; Po.av: Polygonum aviculare; St.me: Stellaria media; Co.ca: Conyza canadensis; So.ol: Sonchus oleraceus; Se.in: Senecio inaequidens*.

LM: Levins’ model; LM + R: Levins’ model with rescue effect; PRM: propagule rain model; PRM + R: propagule rain model with rescue effect.

**Table:**

Street	Species								
	*Po.an*	*La.se*	*Pl.ma*	*Si.ir*	*Ch.al*	*Lo.pe*	*Se.vu*	*Ta.ca*	*Ho.mu*
BARO AIC Alpha	LM + R 95.02 0.006	PRM 54.92	LM 52.1 0.3131	LM 56.11 0.1168	LM 71.8 0.179	PRM 36.21	PRM 53.67	LM + R 95.02 0.006	LM 76.13 0.079
BATA AIC Alpha	PRM 66.66	PRM 18.89	PRM 25.93	PRM 39.34	LM 38.97 0.0523	PRM 19.4	LM 37.6 0.09136	PRM 66.66	PRM 53.75
BERC AIC Alpha	PRM 181	LM 85.19 0.13962	PRM 82.09	LM + R 139.8 0.09	LM 97.95 0.0609	LM 19.37 0.1570	LM 56.63 0.09693	PRM 181	LM 313.3 0.125
BERY AIC Alpha	PRM 46.79	PRM 90	PRM 60.58	LM 115.5 0.09817	LM 116.8 0.05836	LM + R 96.03 0.04925	LM 89.72 0.04786	LM 146.2 0.0934	LM + R 139.1 0.149
CHAR AIC Alpha	LM 207.6 0.102	LM 18.32 0.00125	PRM 47.07	PRM 81.5	LM 99.45 0.1780	PRM 78.21	LM 75.9 0.03562	LM 207.6 0.102	LM + R 177.3 0.198
DAUM AIC Alpha	PRM 261.9	PRM 80.99	PRM 42.77	LM 157.1 0.02945	PRM 109	LM 90.2 0.03485	PRM 116.4	PRM 261.9	PRM 238.8
KESS AIC Alpha	PRM 106.3	LM + R 66.47 0.1492	PRM 33	LM + R 101.2 0.01887	PRM 56.61	PRM 43.03	LM 111.1 0.10049	PRM 106.3	LM 90.38 0.046
LACH AIC Alpha	PRM 55.86	PRM 20.96	LM 28.13 0.5572	PRM 33.8	PRM 28.8	PRM 15.73	PRM 32.21	PRM 55.86	PRM 109.1
MONT AIC Alpha	LM + R 83.86 0.0269	LM 20.61 0.02822	PRM 41.68	LM 47.21 0.05783	PRM 40.91	LM 25 0.000346	PRM 39.51	LM + R 83.86 0.026924	LM 72.28 0.042
POMM AIC Alpha	LM 65.66 0.0022	LM 24 0.1621	PRM 39.02	PRM 30.3	LM 42.03 0.03359	PRM 18.58	LM 28.63 0.003438	PRM 40.01	PRM 36.19
RAPE AIC Alpha	LM 139.3 0.03407	PRM 76.25	PRM 72.41	LM 32.9 0.07050	LM 76.44 0.02267	LM 52.12 0.000627	LM 91.47 0.097210	LM 139.3 0.034077	PRM 238.8
RBER AIC Alpha	LM 196.4 0.001	PRM 95.35	PRM 22.85	LM 61.76 0.03593	PRM 52.38	LM 26.85 0.000601	PRM 84.13	LM 196.4 0.001	PRM 80.77
REUI AIC Alpha	PRM 200.7	LM 34.91 0.1799	PRM 137.5	LM 89.03 0.2841	LM 109.3 0.01652	LM 45.79 0.000244	LM 91.4 0.08372	PRM 200.7	LM 162.1 0.018
TAIN AIC Alpha	PRM 94.59	LM 16.58 0.00185	PRM 23.16	PRM 36.08	PRM 25.81	LM 16.96 0.000256	PRM 27.01	PRM 94.59	LM 1,000 0.001
TERR AIC Alpha	LM 61.94 0.069115	PRM 73.18	LM 49.2 0.0049	PRM 26.15	PRM 56.61	LM + R 58.16 0.000239	PRM 50.95	LM 61.94 0.069115	LM 72.44 0.035
%LM	33.3334	40	20	46.6667	53.3334	46.6667	53.3334	33.3334	40
%LM + R	13.3334	6.6667	0	13.3334	0	13.3334	0	13.3334	20
%PRM	53.3334	53.3334	80	40	46.6667	40	46.6667	53.3334	40

**Table:**

Street	Species									
	*Ca.bu*		*Po.av*	*St.me*	*Co.ca*	*So.ol*	*Se.in*	%LM by street	%LM + R by street	%PRM by street
BARO AIC Alpha	LM 86.93 0.02625		PRM 72.4	LM 54.95 0.135529	PRM 61.93	LM 70.19 0.036	LM 54.92 0.493	53.3334	13.3334	33.3334
BATA AIC Alpha	LM 34.28 0.1357		LM 55.92 0.45078	LM 21.2 0.866361	LM 51.68 0.060558	PRM 40.27	PRM 43.57	40	0	60
BERC AIC Alpha	LM 103.7 0.1494		LM 134.2 0.171844	LM + R 130.2 0.093129	LM + R 118.4 0.154636	PRM 142.1	LM + R 120.3 0.054	46.6667	26.6667	26.6667
BERY AIC Alpha	PRM 116.6		LM 118.3 0.2415	PRM 97.24	LM 116.9 0.320916	PRM 119.9	PRM 39.65	40	13.3334	46.6667
CHAR AIC Alpha	LM + R 136 0.140468		LM 171.1 0.122042	PRM 92.45	LM 141.2 0.209291	LM 70.83 0.08275	PRM 69.8	53.3334	13.3334	33.3334
DAUM AIC Alpha	LM + R 213.2 0.144998		PRM 164.6	PRM 175.9	LM 269.8 0.127495	PRM 120.8	LM 74.71 0.001	26.6667	6.6667	66.6667
KESS AIC Alpha	LM 151.3 0.098562		PRM 33.51	LM 113.5 0.209519	PRM 82.7	LM 122.7 0.0355	PRM 76.73	33.3334	13.3334	53.3334
LACH AIC Alpha	LM 45.48 0.176157		PRM 49.04	LM 32.09 0.067173	LM 141.1 0.212658	PRM 36.11	PRM 22.1	26.6667	0	73.3334
MONT AIC Alpha	LM 42.14 0.033683		PRM 31.78	PRM 50.36	PRM 69.81	PRM 61.85	LM 33.56 0.012	40	13.3334	46.6667
POMM AIC Alpha	PRM 59.35		PRM 37.61	PRM 40.58	PRM 76.55	PRM 41.7	PRM 19.61	26.6667	0	73.3334
RAPE AIC Alpha	PRM 30.28		LM + R 135.2 0.285226	LM + R 134.8 0.05795	PRM 96.66	PRM 142.1	LM 47.13 0.3045	46.6667	13.3334	40
RBER AIC Alpha	PRM 36.96		PRM 92.4	LM + R 129.7 0.011526	LM 220.5 0.030514	LM 145.1 0.05325	PRM 46.85	40	6.6667	53.3334
REUI AIC Alpha	LM 120.4 0.082		LM 136.5 0.19894	LM + R 178.4 0.080947	PRM 262.2	PRM 122	LM 58.43 0.07775	60	6.6667	33.3334
TAIN AIC Alpha	PRM 18.36		PRM 26.75	LM + R 75.47 0.134499	PRM 79.2	PRM 42.63	PRM 15.62	20	6.6667	73.3334
TERR AIC Alpha	LM 46.77 0.077922		PRM 69.97	LM 79.01 0.110642	LM 67.15 0.067623	PRM 73.41	PRM 59.22	46.6667	6.6667	46.6667
%LM by species	53.3334		33.3334	33.3334	46.6667	26.6667	33.3334	Mean LM by street: 40		
%LM + R by species	13.3334		6.6667	33.3334	6.6667	0	6.6667		Mean LM + R by street: 9.3334	
%PRM by species		33.3334	60	33.3334	46.6667	73.3334	60			Mean PRM by street: 50.6667
